# Introduction of the Tea Plant into the United States

**Published:** 1860-10

**Authors:** 


### INTRODUCTION OF THE TEA PLANT INTO THE UNITED STATES.

A few years ago the United States Government was led to take measures for the introduction of the tea plant into the confederation, with a view to establishing, if possible, the preparation of tea in some of the States. The success that had attended Mr. Fortune's operation for the East India Company, having induced them to consult that gentleman, it was finally arranged that he should again proceed to the Celestial Empire, for the purpose of obtaining as abundant a supply of plants for the west as he had secured for the east. His mission has been attended with the most complete success. A minute of the United States Patent Office now before us, states that he was dispatched in March, 1858, that he had obtained seeds enough to plant one hundred acres, a large portion of which had arrived by the 20th of June, 1859, and were flourishing in a propagating house, especially constructed at Washington for their reception. Applications for plants were even then made in such numbers that it had become necessary to refer to Congress for instructions as to their disposal.

Mr. Fortune returned to England some time ago, and has favored us with the following interesting report upon the final result of the expedition:

"It will be seen by the accompanying papers that the results of my expedition to China, for the Government of the United States, have been very satisfactory. In little more than one year about fifty thousand tea plants, and many other vegetable productions useful in the arts, or of an ornamental character,* have been introduced to America from the tea countries of China.

"This success was mainly owing to experience acquired during former visits to these countries. Arriving in China in the month of May, I spent the first few months in visiting numerous tea farms in different parts of the country, where I made arrangements with the natives for large supplies of seeds as soon as they ripened in the autumn. In October and November I repeated my visit to the same districts, and everywhere found supplies of seeds awaiting me. In former

*For example: the Camphor and Tallow trees, Chusan Palm, green dye plant, (Rhamnus,) manure plants, (Trifolium and Coronilla,) Wax-insect tree, (Fraxinus Chinensis,) Yang-mae, (Myrica sp.,) southern fruits, such as the Leechee, Longan, Wampee, &c., &c.

transactions with these tea growers, I had always treated them kindly and liberally, and I now found the advantage that resulted from such treatment. Seeds had been saved for me in all directions; I had only to pick them up and proceed onwards, and was thus enabled to get through a large amount of work in a short space of time. In December I reached the seaport of Shanghae, with the whole of my collections in excellent condition.

"Tea seeds will not retain their vitality long if kept out of the soil. In order to guard against all risk, a large number of Ward's cases had been previously prepared and filled with earth, and to these the seeds were immediately transferred. The first shipment was made in December, a few days after my arrival in Shanghae. Knowing that the vessels would probably arrive in America in the middle or end of March, I thought it likely the seeds would remain in the earth without vegetating during the voyage. Instead, therefore, of sowing the seeds near the surface in the usual manner, I mixed up large quantities with soil, and filled the case up with the mixture of earth and seeds. By this simple plan many thousands of seeds were carried to their destination, and when they arrived they were as sound as if they had been all winter on a Chinese seed-bed. Of course it was necessary to unpack them immediately on arrival, and sow them thinly in other quarters. In the other cases, which were shipped later, this mode of packing would not have been safe. The seeds were therefore sown thickly and covered with earth in the usual manner, and in this state might vegetate on the voyage without any risk whatever. In the one case the object was to get the seeds quickly to their destination without vegetating, for had this taken place, the experiment would have been a failure; in the other they were placed in circumstances favorable for vegetation, and the only change likely to occur would be this, that in China they were only seeds, while towards the end of the voyage or at its termination, they would have changed into healthy young plants.

"The watering, closing the cases, shipping, and last, but not least, securing the good-will of the captain and officers, were all important operations."

Not only has the tea plant been thus introduced to the Eastern States, but it has found its way also to the Western; and our friends on both the Atlantic and Pacific sides are, with their usual energy, setting about growing it.

But there still remains the question whether they can turn it to profitable account. That the climate will be found to suit in some of the vast regions of the West, there can be no reasonable doubt. But merely growing tea plants will not make commercial tea. The difficulty lies in the preparation of it, an operation which, as conducted in China, demands an enormous quantity of labor, the article of which, beyond all others, the United States has the least to spare. But is it really necessary to prepare tea Chinese fashion? to chop it up into little balls and twist it into all sorts of queer shapes with all sorts of names, in order to give it its dietetical value? Surely not. We have ourselves found out that the painted article called green tea is not very wholesome, and by no means the better for the paint; and we quite anticipate that our United States friends will have even already projected some sort of machine that will produce good marketable tea without the assistance of human hands. This, indeed, we know, is the opinion of Mr. Fortune himself.—*London Phar. Jour., from Gardeners Chronicle.*

☞ A death by chloroform is this week reported in our columns from Bellevue Hospital. The anæesthetic was administered by a junior member of the house staff, with as much care as is usual in hospital practice, and commendable exertions were made to resuscitate the patient. The post mortem examination revealed fatty degeneration of the heart, an apoplectic condition of both lungs, and slight evidences of former inflammation of the meninges of the brain. This is the second case of death by chloroform which has occurred in that hospital within a twelvemonth. Considering the daily use of anæesthetics in this large hospital, and the immense aggregate of cases of its employment in a year, and above all the careless manner in which it is often administered, this exemption from fatal consequences is truly remarkable. We learn that this case has raised the question in the Medical Board of the propriety of using chloroform in the practice of that hospital. We believe that this agent was long since excluded from the New York, Pennsylvania, and Massachusetts General Hospitals.—*American Medical Journal.*

